# Multivariate analysis of the influence of peri-implant clinical parameters and local factors on radiographic bone loss in the posterior maxilla: a retrospective study on 277 dental implants

**DOI:** 10.1007/s00784-020-03666-x

**Published:** 2020-11-05

**Authors:** Mariane B. Sordi, Vittoria Perrotti, Flavia Iaculli, Keila C. R. Pereira, Ricardo S. Magini, Stefan Renvert, Stefano Antonio Gattone, Adriano Piattelli, Marco A. Bianchini

**Affiliations:** 1 Center for Research on Dental Implants, Federal University of Santa Catarina, Florianópolis, Brazil; 2 Department of Medical, Oral and Biotechnological Sciences (DSMOB), University of Chieti-Pescara, Via dei vestini, 31, 66100 Chieti, Italy; 3 Department of Neuroscience and Reproductive and Odontostomatological Sciences, University of Naples Federico II, Naples, Italy; 4 Public Health Sciences, University of South of Santa Catarina, Tubarao, Brazil; 5 Perio/Implantology, Department of Dentistry, Center for Research on Dental Implants, Federal University of Santa Catarina, Florianópolis, Brazil; 6 Oral Health Sciences, Kristianstad University School of Dentistry, Kristianstad, Sweden; 7 Dublin Dental Hospital, Trinity College, Dublin, Ireland; 8 Blekinge Institute of Technology, Karlskrona, Sweden; 9 Department of Philosophical, Pedagogical and Economic-Quantitative Sciences (DiSFPEQ), University of Chieti-Pescara, Chieti, Italy; 10 Biomaterials Engineering, Catholic University of Murcia (UCAM), Murcia, Spain; 11 Villaserena Foundation for Research, Città Sant’Angelo (Pescara), Italy; 12 Perio/Implantology, Department of Dentistry, Center for Research on Dental Implants, Federal University of Santa Catarina, Florianópolis, Brazil

**Keywords:** Bone loss, Dental implant, Local factors, Peri-implantitis, Posterior maxilla

## Abstract

**Objectives:**

The aim of the present study was to investigate whether peri-implant clinical parameters (modified plaque index (mPI), bleeding and/or suppuration on probing (B/SOP)) and local factors (type of prostheses, screw emergence, platform diameter, and abutment angulation) might contribute to the development of additional bone loss and peri-implantitis around dental implants.

**Materials and methods:**

Two hundred seventy-seven external hex connection implants placed in the posterior maxilla of 124 patients were retrospectively evaluated. They were divided into two groups: physiologic bone loss < 2 mm (PBL) or additional bone loss ≥ 2 mm (ABL). GEE logistic regression was applied to evaluate the influence of type of prostheses (implant-supported single crown (ISSC), fixed partial denture (ISFPD), and full denture (ISFD)) and clinical parameters (mPI and S/BOP) on bone loss.

**Results:**

Among the 277 implants, 159 (57.4%) presented PBL and 118 (42.6%) presented ABL. Within the ABL group, 20.6% implants were diagnosed with peri-implantitis. mPI significantly correlated with the type of prosthesis and the highest value of mPI (index = 3) was observed in ISFD (23.8%). Moreover, peri-implantitis was more frequently associated with ISFD (32.79%) than ISSC and ISFDP (13.79% and 13.48, respectively)

**Conclusions:**

ISFD in the posterior maxilla presented high rates of ABL and showed a higher prevalence of peri-implantitis. None of the local factors seemed to contribute to the development of these conditions. Further investigations are needed to prospectively support the results of the present study.

**Clinical relevance:**

Patients rehabilitated with ISFD should be carefully monitored and have more frequent maintenance visits to prevent or control peri-implant bone loss.

## Introduction

Peri-implant bone loss has a multifactorial pathogenesis and it is linked to a multitude of risk factors related to the dental implant (i.e., surface modifications, position, type of prosthesis, implant-abutment connection, timing of loading), to the patient (i.e., systemic and local factors), and to the clinician (i.e., technical skills) [1, 2]. Patient-related systemic factors mainly include the previous history of periodontitis, diabetes, and smoking habits, while local factors mostly involve parafunctional habits, bone quality and quantity, and poor plaque control [1, 3].

A controlled bone loss 1-year post-loading (less than 1.5 mm) and an additional 0.2 mm yearly after the first year of function has been generally accepted [4, 5]; however, additional values of bone loss should be investigated in depth. The gradual loss of marginal bone after osseointegration, without a bacterial infection able to cause bleeding and/or suppuration, can be identified as late or additional bone loss (ABL) [6]. ABL was reported to be initiated and maintained over time by iatrogenic factors or local conditions, such as occlusal trauma, implant features, and prosthetic restorations [4, 7, 8]; however, in the presence of ABL along with inflammation of the peri-implant connective tissues (i.e., bleeding and/or suppuration), peri-implantitis can be claimed [3]. The clinical diagnosis of peri-implantitis remains a controversial issue since the absence of univocal diagnostic criteria and specific thresholds [9]; specifically, it has been reported that the determination of a physiological probing depth (PD) at implant sites is difficult [3] and that PD and bleeding on probing (BOP) did not seem to be correlated with the mean bone loss [9]. In addition, bone loss is usually evaluated on radiographs where a difference of about 1–2 mm could be purely assigned as inter-examiner different assessments[10]. Therefore, peri-implantitis is an inappropriate term to describe all the cases of crestal bone loss [11], although progressive crestal bone loss around implants in the absence of clinical signs of soft tissue inflammation is a rare event [3].

The impact of the type of implant-supported prostheses on peri-implant bone loss and peri-implantitis remains unclear [12, 13]. There is also a need for a deeper understanding of the role of prosthetic-related local factors, contributing to the development of peri-implant bone loss [13, 14]. The question whether platform-matched implants are more at risk for failure and loss of marginal bone than platform-switched implants has received increasing attention in the last years. A recent meta-analysis (2015) by Chrcanovic et al. [15] suggested that there is a significantly less MBL at implants with platform switching than on implants with platform matching; this difference increase with the increase of the follow-up time and of the mismatch between the implant platform and the abutment. Another hot topic is the one abutment one-time workflow. Indeed, definitive abutments placed at implant insertion and never removed might be a critical strategy to ensure minimal disruption to the peri-implant hard and soft tissues and to preserve marginal bone level, although further RCTs with longer follow-up are needed to better understand the clinical significance of such approach [16, 17].

The anatomical and morphological structure of the upper jaw, which has a lower density and a reduced bone volume and may consequently undergo a high degree of alveolar ridge resorption, is considered to be critical to the success of dental implants [18–21]; furthermore, the heavy masticatory load in the posterior regions might also influence the success rate in this area [20]. Vervaeke et al. [22] reported high values of peri-implant bone loss in smokers and in the maxilla, corroborating the scientific literature showing that maxillary implants generally present more severe or more frequent cases of peri-implantitis than implants placed in the mandible [13, 20, 23, 24].

Therefore, the aim of the present study was to evaluate whether peri-implant clinical parameters (modified plaque index (mPI), bleeding, and/or suppuration on probing (B/SOP)) as well as local factors, such as type of prostheses, screw emergence, platform diameter, and abutment angulation might contribute to the development of ABL and eventually peri-implantitis around dental implants in the posterior maxilla.

## Material and methods

### Sample selection

The present study was undertaken at the periodontal clinic of the center of research and continuing education at the Federal University of Santa Catarina (UFSC), Brazil. All patients signed a consent form authorizing data collection, following approval by the Ethics Committee on Human Research of the UFSC, Brazil (approval number: 1.430.035). The study was conducted in accordance with the Helsinki Declaration of 1975, as revised in 2004.

One hundred twenty-four patients, who were treated with titanium dental implants from March 2000 to March 2010 and were rehabilitated with implant-supported fixed prostheses in the Center of Research in Dental Implants (CEPID) of the Health Sciences Center of the same university, were enrolled in the present study.

Only the implants installed in the posterior region of the maxilla were included in the study to specifically assess the peri-implant bone loss associated with the heavy masticatory load that occurs in the posterior region. Patients with incorrect and/or incomplete medical records and requiring grafting or sinus lift prior to implant placement, implants with an inadequate distance between the teeth and implants (tooth-implant distance < 1.5 mm, inter-implant distance <3 mm) [25, 26], and poorly handled or distorted periapical radiographs were all excluded. Supportive periodontal/peri-implant therapy after prosthesis installation was performed according to the individual’s demands.

All implants were analyzed at least 12 months after prosthesis installation. Data related to the prostheses, such as type of prostheses, screw emergence, platform diameter, and abutment angulation were collected. Specifically, the type of prostheses was divided into implant-supported single crown (ISSC), implant-supported fixed partial denture (ISFPD), implant-supported full denture (ISFD). The screw emergence, defined as the location of the screw access regarding the occlusal face of prosthetic crowns, was divided into ideal (centralized), buccally or lingually oriented. Finally, platform diameter was classified as narrow: 3.3, regular: 4.1, or wide: 5.0 and abutment angulation as 0° or 17°. Metal-ceramic crowns were used to develop single crowns and partial dentures, and metal-acrylic dentures were used in the case of full-arch rehabilitations. Only screw-retained prostheses were performed and included in the study. To perfectly recreate the patient’s occlusion, prostheses were developed according to the position of casts on the articulator and further verified by interocclusal records to ensure correct intercuspation and maintenance of the vertical dimension of occlusion [27].

**Fig. 1:**
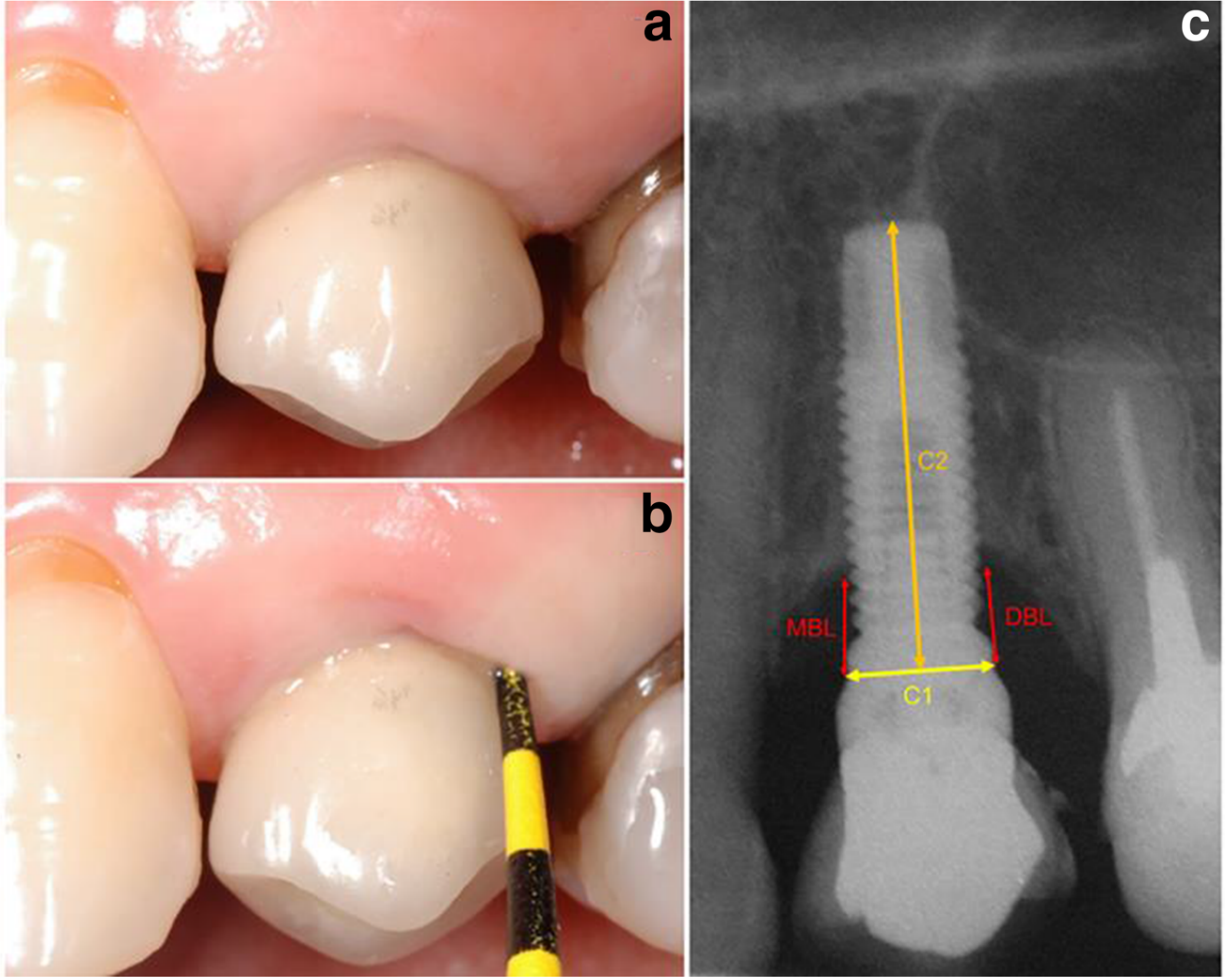
**a** Clinical view of an implant-supported single crown (ISSC). **b** Probing depth measured around an implant-supported single crown (ISSC). **c** Peri-implant bone loss was measured on both sides of each implant platform (DBL, distal bone loss; MBL, mesial bone loss) as the distance between the implant-abutment interface and the first bone-to-implant contact. To correct the dimensional distortion in the radiograph, the software was calibrated according to the true implant diameter (C1) and length (C2)

**Fig. 2:**
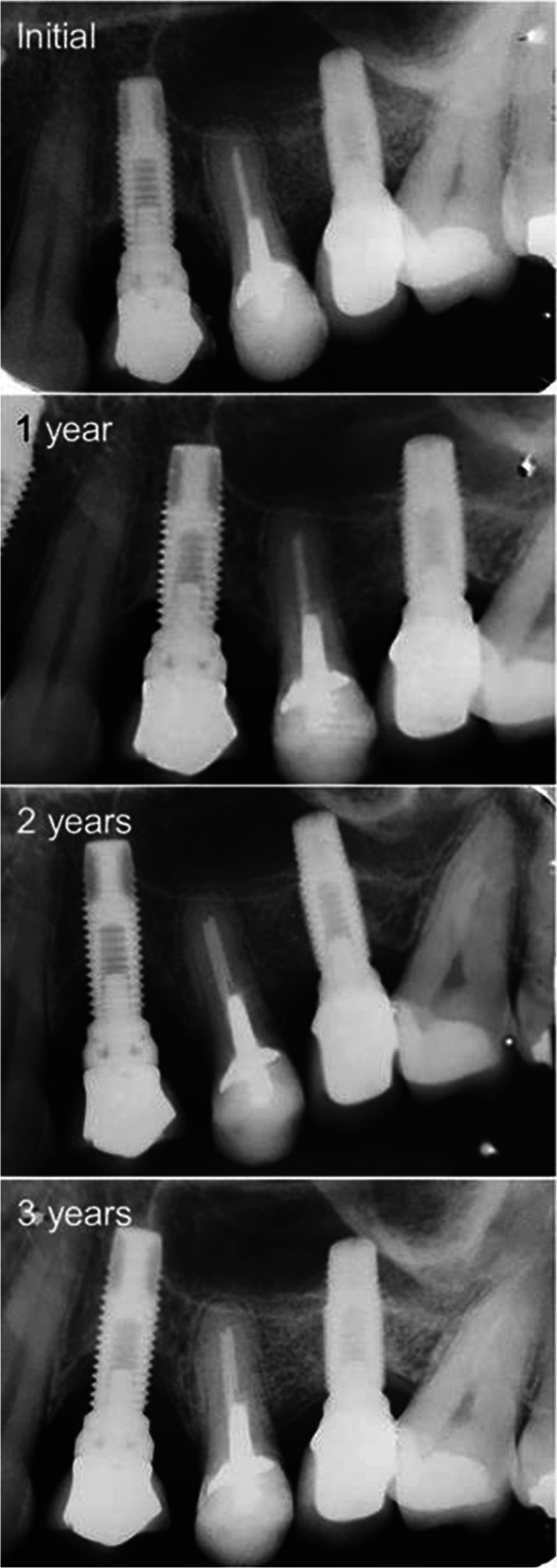
Radiographic images of two implant-supported single crowns (ISSC) at the 3-year follow-up

The following clinical parameters were measured by a calibrated periodontist to reduce the intra-examiner error (*k* > 0.75) using a millimeter periodontal probe (PCV12PT, Hu-Friedy Inc, Chicago, IL, USA) (Fig. 1 a and b) and when necessary, the screw-retained prostheses were removed:

- Presence of bacterial plaque according to modified plaque index (mPI) [28], in four sites per implant (mesial, buccal, distal, and lingual): score 0–3. Only the highest value per implant was recorded;
- Bleeding or suppuration upon probing (B/SOP): number of sites out of four (mesial, buccal, distal, and lingual) per implant with positive bleeding or suppuration up to 30 s after probing. Probing was gently conducted parallel to the long axis of the dental implant to avoid false positive. Probing pressure was standardized during periodontist calibration [29, 30]. Moreover, radiographic bone loss (RBL) was measured on intraoral periapical radiographs taken by the mean of parallel cone technique using a Rinn alignment system (Insight Film Kodak, Carestream, Rochester, NY, USA) with a rigid film-object x-ray source coupled to a beam-aiming device to achieve reproducible exposure geometry [31]; an acrylic radiographic stent was customized for each implant allowing the patient to bite in the same position during the follow-up visits. After digital scanning of each radiograph, the measurements were performed on both sides of each implant platform (mesial and distal) by a previously calibrated single examiner (RSM) using an image analysis software (version 3.7.0 Digimizer, Medical Software Brolkstraat, Belgium). In order to determine the intra-examiner reproducibility, repeated measures were performed with a 7-day interval (*k* > 0.75). The means of the two measurements (0 and 7 days) were calculated and the highest bone loss value was established for each implant. To correct the dimensional distortion in the radiograph, the software was calibrated with the true implant diameter and length. The values were obtained as modifications in the distance between the implant platform and the first radiographic bone contact with the implant on both the mesial (MBL) and on the distal (DBL) sides (Fig. 1c). According to the amount of bone loss, implants were divided into implants presenting physiologic bone loss (PBL) when < 2 mm or additional bone loss (ABL) when ≥ 2 mm, as proposed by Souza et al. (2013) [6]. The association of ABL with B/SOP was considered peri-implantitis [4–6, 32, 33], while the presence of PBL with B/SOP was classified as peri-implant mucositis [34]. Each radiograph was taken at the moment of the prosthesis’s installation and every year during the follow-up visit (Fig. 2).

### Statistical analysis

Since the data are clustered, i.e., with repeated measurements (number of implants) for each patient, generalized estimating equations (GEE) in a logistic regression setting provide a good way to model the data. This technique takes into account the correlation within the cluster during the analysis [35, 36]. Statistical analysis was performed using the Statistical Software R (version3.6.2). To assess whether there were differences in the proportion of patients with ABL according to the explanatory variables, the GEE method was applied using the R package gee [37]. When the response variables were mPI and BOP, the multinomial GEE logistic regression model was applied using the R package multgee [38]. The GEE logistic regression models were fitted by specifying an “unstructured” working correlation structure. Odds ratios and 95% confidence intervals (CI) based on robust standard errors were computed. The Wald test based on solid standard error was used to assess the significance of each factor.

Firstly, the univariate effect of each factor related to local bone loss was analyzed; then, the effect of local factors on mPI and BOP was evaluated and finally, the impact of the type of prosthesis when controlled for mPI and BOP was assessed.

## Results

A total of 124 patients (43 men and 81 women, mean age 51 years) and 277 screw-retained external hex connection dental implants (219 Conexão, São Paulo, SP, Brazil; 32 SIN, São Paulo, SP, Brazil; 26 Neodent, Curitiba, PR, Brazil) (mean 2.23 implants/patient) were placed in the posterior maxilla and distributed as reported in Table 1. Among the 124 patients, 21 (16.93%) presented periodontal disease, 9 (7.26%) were smokers, and 12 (9.68%) were ex-smokers. Regarding medical condition, 25 (20.15%) individuals presented arterial hypertension, 11 (8.87%) patients had diabetes, 5 (4.03%) participants revealed osteoporosis, and 5 (4.03%) individuals were previously submitted to radio or chemotherapy to treat cancer.

**Table 1:** Implant distribution according to their location in the posterior maxilla

Implant location	Frequency
First premolar	106 (38.27%)
Second premolar	85 (30.68%)
First molar	73 (26.35%)
Second molar	13 (4.7%)

The mean observation period for all restorations was 37.38 months ± 22.52 (range: 12.02–100.76 months). The frequency and percentage distribution of implants per patient are depicted in Table 2. Among the 277 implants, 159 (57.4%) presented PBL and 118 (42.6%) presented ABL. GEE logistic regression showed that a significantly greater probability to develop ABL (OR: 3.149; 95% CI: 1.256–7.895) was found in implants with mPI 3 (Table 3). Within the ABL group, a total of 57 implants (20.6%) showed also BOP and were diagnosed as implants with peri-implantitis. A significantly greater chance to develop ABL was detected only with BOP 2 (OR: 1.996, 95% CI: 1.022–3.899) (Table 3). None of the analyzed implants showed suppuration upon probing (SOP). Data regarding the peri-implant mean bone loss (mm) according to the different follow-up interval are reported in Appendix Table 14.

**Table 2:** Frequency and percentage distribution of implants per patient

Number of implants per patient	Patients *n* (%)
1	46 (37.09%)
2	42 (33.87%)
3	16 (12.90%)
4	7 (5.64%)
5	8 (6.45 %)
6	4 (3.22%)
7	1 (0.80%)

**Table 3:** Frequency and percentage distribution of implants with PBL and ABL according to mPI score and related GEE logistic regression; frequency and percentage distribution of implants with PBL and ABL according to BOP and related GEE logistic regression

	PBL	ABL	Odds ratio	*P* value	95% CI
mPI					
0	123 (62.8%)	73 (37.2%)			
1	17 (50%)	17 (50%)	1.923	0.101	0.880–4.199
2	8 (50%)	8 (50%)	1.155	0.793	0.394–3.387
3	11 (35.5%)	20 (64.5%)	3.149	0.014*	1.256–7.895
BOP					
0	109 (64.1%)	61 (35.9%)			
1	27 (50.9%)	26 (49.1%)#	1.443	0.245	0.776–2.683
2	18 (41.9%)	25 (58.1%)#	1.996	0.043*	1.022–3.899
3	5 (45.5%)	6 (54.5%)#	1.716	0.379	0.516–5.699

#ABL associated with BOP was diagnosed as peri-implantitis

*Statistically significant

Considering the type of implant-supported prostheses, implants rehabilitated with both ISSC (total number: 87; 31.4%) and ISFPD (total number: 89; 32.1%) showed more frequently PBL (63 and 61 implants, respectively) than ABL (24 and 28 implants, respectively); on the other hand, ISFD (total number: 101; 36.5%) was more often associated with ABL (66 implants) than PBL (35 implants). ABL was 1.745 (OR) time more likely in ISFD than ISSC (*P* value < 0.001, 95% CI: 2.828–11.601) and 4.450 (OR) than in ISFD than ISFPD (*P* value < 0.001, 95% CI: 2.173–9.111). Screw emergence, platform diameter, and abutment angulation showed no significant association with bone loss (Tables 4 and 5).

**Table 4:** Frequency and percentage distribution of implants with PBL and ABL regarding the type of prosthesis, screw emergence, platform diameter, abutment angulation, and related GEE logistic regression

Type of prostheses	PBL	ABL	Odds ratio	95% CI	*P* value
Implant-supported single crown	63 (72.4%)	24 (27.6%)			
Implant-supported fixed partial denture	61 (68.5%)	28 (31.5%)	0.631	0.631–2.624	0.487
Implant-supported dull denture	35 (34.7%)	66 (65.3%)	1.745	2.828–11.601	< 0.001*
Screw emergence					
Ideal	139 (57.4%)	103 (42.6%)			
Buccally	18 (58.1%)	13 (41.9%)	1.976	0.484–2.390	0.858
Lingually	2 (50%)	2 (50%)	1.436	0.213–9.674	0.710
Platform diameter					
Regular	142 (56.6%)	109 (43.4%)			
Narrow	5 (55.6%)	4 (44.4%)	1.264	0.318–5.020	0.740
Large	12 (70.6%)	5 (29.4%)	0.394	0.122–1.274	0.230
Abutment angulation					
0°	148 (57.6%)	109 (42.4%)			
17°	11 (55%)	9 (45%)	1.002	0.38–2.643	0.997

*Statistically significant

**Table 5:** GEE logistic regression : type of prostheses - implant-supported single crown (ISSC), implant-supported fixed partial denture (ISFPD), implant-supported full denture (ISFD) - in comparison with PBL and ABL

Type of prostheses effect	Odds ratio	*P* value	OR (95% CI)
ISSC-ISFPD	0.631	0.487	0.631–2.624
ISSC-ISFD	1.745	< 0.001*	2.828–11.601
ISFPD-ISFD	4.450	< 0.001*	2.173–9.111

*Statistically significant

Frequency and percentage distribution of implants with mPI 0, 1, 2, and 3, according to the type of prosthesis, screw emergence, platform diameter, and angulation, are shown in Table 6. After GEE logistic regression, it was found that the type of prosthesis showed a statistically significant effect on mPI. Specifically, significantly higher values of mPI (2 and 3) had a high association with implants rehabilitated with FD (9.9% and 23.8%, respectively) and mPI 2 and 3 were respectively 4.640 (OR) and 11.309 (OR) more likely in ISFD than in ISSC (mPI 2: *P* value 0.038; 95% CI: 1.091–19.738; mPI 3: *P* value 0.008; 95% CI: 1.897–67.413) (Table 7). Besides, both lingual and buccal screw emergency had a significant impact on the mPI 1, 2, and 3 either when compared to mPI 0 or to mPI 1 (Table 8). Abutment angulation did not affect mPI.

**Table 6:** Frequency and percentage distribution of implants with mPI 0, 1, 2, and 3 according to the type of prosthesis, screw emergence, platform diameter, and angulation

mPI	0	1	2	3
Type of prostheses				
Implant-supported single crown	69 (79.3%)	11 (12.6%)	4 (4.6%)	3 (3.4%)
Implant-supported fixed partial denture	74 (83.1%)	9 (10.1%)	2 (2.2%)	4 (4.5%)
Implant-supported full denture	53 (52.5%)	14 (13.9%)	10 (9.9%)	24 (23.8%)
Screw emergence				
Ideal	171 (70.7%)	32 (13.2%)	11 (4.5%)	28 (11.6%)
Buccally	24 (77.4%)	1 (3.2%)	4 (12.9%)	2 (6.5%)
Lingually	1 (25%)	1 (25%)	1 (25%)	1 (25%)
Platform diameter				
Regular	183 (72.9%)	25 (10%)	14 (5.6%)	14 (5.6%)
Narrow	4 (44.4%)	5 (55.6%)	0 (0%)	0 (0%)
Large	9 (52.9%)	4 (23.5%)	2 (11.8%)	2 (11.8%)
Abutment angulation				
0°	185 (72%)	30 (11.7%)	13 (5.1%)	29 (11.6%)
17°	11 (55%)	4 (20%)	3 (16%)	2 (10%)

**Table 7:** GEE multinomial logistic regression of implants with mPI 0, 1, 2, and 3 regarding the type of prosthesis

Response variable mPI	Type of prostheses effect	Odds ratio	*P* value	OR (95% CI)
0–3	ISSC-ISFPD	2.164	0.343	0.441–10.655
	ISSC-ISFD	11.309	0.008*	1.897–67.413
0–2	ISSC-ISFPD	0.571	0.578	0.079–4.107
	ISSC-ISFD	4.640	0.038*	1.091–19.738
0–1	ISSC-ISFPD	0.968	0.947	0.366–2.562
	ISSC-ISFD	2.408	0.111	0.816–7.106

*Statistically significant

**Table 8:** GEE multinomial logistic regression of implants with mPI 0, 1, 2, and 3 regarding the screw emergence

Response variable mPI	Screw emergence	Odds ratio	*P* value	OR (95% CI)
0–3	I-B	1.289	0.293	0.803–2.068
	I-L	2.722	0.092	0.849–8.728
0–2	I-B	2.322	0.068	0.939–5.743
	I-L	14.345	0.020*	1.513–136.03
0–1	I-B	0.197	0.035*	0.043–0.895
	I-L	4.905	0.142	0.587–40.954
1–3	I-B	6.487	0.019*	1.352–31.119
	I-L	0.558	0.420	0.135–2.306
1–2	I-B	11.685	0.019*	1.487–91.834
	I-L	2.926	0.375	0.273–31.295

*Statistically significant

Table 9 shows the frequency and percentage distribution of implants with BOP 0, 1, 2, and 3 according to the type of prosthesis, screw emergence, platform diameter, and angulation. Only type of prosthesis revealed significant impact with BOP; specifically, ISFPD when compared to ISSC had 8.782 more chance to have BOP 3 when the base outcome was BOP 0 and 20.658 when the base outcome was BOP 1 (Table 10).

**Table 9:** Frequency and percentage distribution of implants with BOP 0, 1, 2, and 3 according to the type of prosthesis, screw emergence, platform diameter, and angulation

BOP	0	1	2	3
Type of prostheses				
Implant-supported single crown	51 (58.6%)	23 (26.4%)	12 (13.8%)	1 (1.1%)
Implant-supported fixed partial denture	62 (69.7%)	10 (11.2%)	11 (12.4%)	6 (6.7%)
Implant-supported full denture	57 (56.4%)	20 (19.8%)	20 (19.8%)	4 (4%)
Screw emergence				
Ideal	149 (61.6%)	47 (19.4%)	36 (14.9%)	10 (4.1%)
Buccally	20 (64.5%)	5 (16.1%)	6 (19.4%)	0 (6.7%)
Lingually	1 (25%)	1 (25%)	1 (25%)	1 (25%)
Platform diameter				
Regular	155 (61.8%)	49 (19.5%)	37 (14.7%)	10 (4%)
Narrow	3 (33.3%)	2 (22.2%)	3 (33.3%)	1 (11.1%)
Large	12 (70.6%)	2 (11.8%)	3 (17.6%)	0 (0%)
Abutment angulation				
0°	161 (62.6%)	46 (17.9%)	39 (15.2%)	11 (4.3%)
17°	9 (45%)	7 (35%)	4 (20%)	0 (0%)

**Table 10:** GEE multinomial logistic regression of implants with BOP 0, 1, 2, and 3 regarding the type of prosthesis

Response variable BOP	Type of prostheses effect	Odds ratio	*P* value	OR (95% CI)
0–3	ISSC-ISFPD	8.782	0.038*	1.132–68.152
	ISSC-ISFD	4.934	0.215	0.396–61.453
0–2	ISSC-ISFPD	0.975	0.958	0.384–2.478
	ISSC-ISFD	1.677	0.307	0.622–4.522
0–1	ISSC-ISFPD	0.425	0.060	0.174–1.037
	ISSC-ISFD	0.831	0.683	0.341–2.022
1–3	ISSC-ISFPD	20.658	0.038*	2.600–164.115
	ISSC-ISFD	5.938	0.215	0.469–75.223

*Statistically significant

Finally, considering the association between the type of prosthesis and the occurrence of peri-implantitis, it was found that 7 implants (2.53%) rehabilitated with ISSC presented both mPI and ABL, 5 implants (1.8%) rehabilitated with ISFPD presented both mPI and ABL, and 33 implants (11.91%) supporting ISFD presented both mPI and ABL (Table 11). Accordingly, considering the association between the type of prosthesis and the occurrence of peri-implantitis, it was found that 12 implants (4.3%) rehabilitated with ISSC presented both BOP and ABL, 12 implants (4.3%) rehabilitated with ISFPD presented BOP and ABL, and 33 implants (11.9%) supporting FD presented BOP and ABL (Table 12). GEE multivariate logistic regression regarding mPI, BOP, and type of prosthesis in comparison with PBL and ABL corroborates the hypothesis that peri-implantitis is significantly associated with ISFD (*P* < 0.001; 95% CI: 2.301–11.035) (Table 13).

**Table 11:** Frequency and percentage distribution of implants with mPI 0, 1, 2, and 3

Types of prostheses	mPI	PBL	ABL
Implant-supported single crowns (87)	0	52 (75.4%)	17 (24.6%)
	1	7 (63.6%)	4 (36.4%)*#*
	2	3 (75%)	1 (25%)*#*
	3	1 (33.3%)	2 (66.7%)*#*
Implant-supported fixed partial denture (89)	0	51 (68.9%)	23 (31.1%)
	1	4 (44.4%)	5 (55.6%)*#*
	2	2 (100%)	0 (0)*#*
	3	4 (100%)	0 (0)*#*
Implant-supported full dentures (101)	0	20 (37.7%)	33 (62.3%)
	1	6 (42.9%)	8 (57.1%)*#*
	2	3 (30%)	7 (70%)*#*
	3	6 (25%)	18 (75%)*#*

#ABL associated with BOP was diagnosed as peri-implantitis

**Table 12:** Bleeding on probing (BOP) 0, 1, 2, and 3 showing PBL and ABL according to the type of prosthesis

Types of prostheses	BOP	PBL	ABL
Implant-supported single crowns (87)	0	39 (76.5%)	12 (23.5%)
	1	16 (69.6%)	7 (30.4%)#
	2	8 (66.7%)	4 (33.3%)#
	3	0 (0%)	1 (100%)#
Implant-supported fixed partial denture (89)	0	46 (74.2%)	16 (25.8%)
	1	7 (70%)	3 (30%)#
	2	5 (45.4%)	6 (54.5%)#
	3	3 (50%)	3 (50%)#
Implant-supported full dentures (101)	0	24 (42.1%)	33 (57.9%)
	1	4 (20%)	16 (80%)#
	2	5 (25%)	15 (75%)#
	3	2 (50%)	2 (50%)#

#ABL associated with BOP was diagnosed as peri-implantitis

**Table 13:** GEE multivariate logistic regression: mPI, BOP, and type of prosthesis in comparison with PBL and ABL

Type of prostheses	Odds ratio	*P* value	95% CI
Implant-supported single crown			
Implant-supported fixed partial denture	1.343	0.445	0.631–2.862
Implant-supported Full Denture	5.039	< 0.001*	2.301–11.035
mPI			
0			
1	1.293	0.444	0.669–2.498
2	1.891	0.081	0.924–3.868
3	1.667	0.439	0.457–6.082
BOP			
0			
1	1.453	0.451	0.550–3.835
2	0.703	0.574	0.205–2.406
3	1.368	0.626	0.387–4.845

*Statistically significant

## Discussion

The posterior region of the maxilla is known to present low bone density and be subjected to heavy masticatory loads [20], which might be associated with ABL. Indeed, French et al. [39] retrospectively reported cases of implants placed in the posterior mandible and posterior maxilla with equivalent crestal bone levels at the baseline; however, the bone loss percentage was found to increase at a faster rate in the posterior maxilla. Moreover, Noda et al. [40] stated that the placement of dental implants in the posterior maxilla should be considered as a risk factor for late implant failure. The maxillary location of dental implants had been even identified as a statistically significant risk indicator for the development of peri-implantitis [41]. Francetti et al. [42] reported that the cumulative proportion of implants with peri-implantitis was significantly higher in the maxilla than in mandible after a 10-year observation period. In the present study, a total of 57 out of 277 implants (20.6%) presented peri-implantitis, corroborating the results of a recent systematic review, which found an estimated weighted mean prevalence of peri-implantitis of 22% (14–30%) [39, 43]. None of the analyzed implants showed suppuration upon probing. Nevertheless, consensus statements indicate that suppuration is a common finding at sites diagnosed with peri-implantitis [44]. In addition, other consensus conferences defined peri-implantitis as “infection with suppuration associated with clinically significant crestal bone loss” [11]. Based on this definition, a 10-year clinical prospective study showed low incidences of peri-implantitis [45], while a more recent study [46] detected suppuration in 30.16% of peri-implantitis diagnosed patients, corresponding to 17.39% implants with suppuration.

Regarding the type of prostheses, there are disagreements concerning the different behavior between single-implant prostheses and multiple retained prostheses supported by dental implants [12, 13]. Nevertheless, the present study revealed significant differences between the type of prostheses for mPI values of 2 and 3 and demonstrated that the ABL was lower in the presence of ISSC than ISFPD and ISFD. Indeed, the impact of ISFD on the bone loss was significantly higher than the one of ISFPD and ISSC, corroborating the hypothesis that implants rehabilitated with ISFD had a significantly greater chance of bone loss and peri-implant inflammation than ISSC or ISFPD [13, 47]. This data were further supported by GEE multivariate logistic regression regarding mPI, BOP, and type of prosthesis in comparison with PBL and ABL showing a significant association to ISFD (*P* < 0.001; 95% CI: 2.301–11.035). Indeed, besides the higher prevalence of bone loss in ISFD, this type of prostheses also presented higher mPI, probably due to cleaning-related issues, which in turn might contribute to ABL, as observed in the present study. There is considerable evidence indicating that plaque accumulation is the main etiological factor for peri-implant soft tissue inflammation [7, 48, 49]; however, a previous history of periodontal disease and full-mouth rehabilitation was identified as risk factors for peri-implantitis [13]; therefore, the type of prosthesis should be carefully chosen during the treatment plans of patients at risk of peri-implantitis. Accordingly, a significantly greater chance to develop ABL was detected in implants with BOP 2, but unexpectedly not with BOP 3; this might be due to the limited number of cases associated with BOP 3 and evaluated in the present study. Indeed, only 11 implants showed BOP 3, so the sample size was very low compared to the number of implants [48] showing BOP 2.

Finally, it is important to relate local factors, such as screw emergence, implant platform diameter, and angulation of the prosthetic abutment to bone loss as these variables can be controlled in an implant-supported rehabilitation [13, 48]. However, our data show that they did not impact on both mPI and BOP apart from the screw emergence that either in its lingual or in its buccal position showed more chance of ABL.

Indeed, peri-implantitis has recently been categorized in plaque induced, surgically and prosthetically triggered and it has been shown that they are different entities associated with distinguishing predictive profiles. A deep knowledge of the risk factors can drive the implant treatment planning but also the clinical decision-making to solve complications and, last but not least, the identification of specific factors involved in peri-implantitis onset might be the essential precondition for treatment success [50]. Hence, the importance of the present study in searching for a relation between peri-implant clinical parameters and local factors potentially affecting bone loss in order to orient clinicians toward the appropriate causal treatment approach.

The present clinical results should be read considering that this is a retrospective evaluation and that some of the enrolled patients, who presented periodontal disease, smoking habits, hypertension, diabetes, osteoporosis and cancer, were not withdrawn from the sample and, somehow, could have affected the results. This latter point has not been verified in the present study with correlation analyses due to the small number of patients affected by systemic diseases, which would have not led to achieve any statistical significance regarding an eventual impact of systemic diseases on ABL and or peri-implantitis.

Therefore, prospective studies with clinical and radiological baseline data that reflect the status after initial healing and remodeling, with an appropriate sampling frame, adequate sample size and sampling method are needed to draw final conclusions on the effects of local factors on the incidence of peri-implantitis. Finally, only hexagonal platform connections were included in the present study, although there is a current trend to install platform switching or Morse taper connections. Indeed, platform-switched implants have been shown to decrease bone loss compared with non-platform-switched implants since there is no abutment connection near the crestal bone [51]; therefore, a similar study analyzing only conical connections in the posterior maxilla might be interesting.

Further randomized and controlled trials are needed to support the results of the present study.

## Conclusions

Within the limitation of the present retrospective study, it could be concluded that half of the implants presenting ABL were diagnosed with peri-implantitis. Moreover, peri-implant inflammation, ABL, and peri-implantitis showed a higher prevalence in implants restored with ISFD, due to their greater association with high index mPI. Therefore, patients rehabilitated with ISFD should be carefully monitored and have more frequent maintenance visits to prevent or control peri-implant bone loss.

## Appendix

**Table 14:** Peri-implant mean bone loss (mm) according to the different follow-up interval. *SD*, standard deviation; *n*, number of evaluated implants within the follow-up period

	Bone loss	SD	*n*
1–2 years	1.97054098	0.947634	122
2–3 years	1.77955556	0.954065	36
3–4 years	2.11842553	1.061084	47
4–5 years	2.35384211	1.022692	19
5–6 years	2.84684375	1.458391	32
6–7 years	2.58392857	1.155898	14
7–9 years	1.99357143	1.47945	7
Mean	2.23524399	1.154173	277
